# Prognostic factors associated with failure of total elbow replacement: a protocol for analysis of National Joint Registry data in England

**DOI:** 10.1136/bmjopen-2025-098729

**Published:** 2025-07-01

**Authors:** Zaid Hamoodi, Adrian Sayers, Michael R Whitehouse, Lianne Kearsley-Fleet, Jamie C Sergeant, Adam C Watts

**Affiliations:** 1 Centre for Epidemiology Versus Arthritis, Manchester Academic Health Science Centre, The University of Manchester, Manchester, UK; 2 Upper Limb Unit, Wrightington Wigan and Leigh NHS Foundation Trust, Wigan, UK; 3 Muscloskeletal Research Unit, University of Bristol, Bristol, UK; 4 University Hospitals Bristol NHS Foundation Trust and University of Bristol, NIHR Bristol Biomedical Research Centre, Bristol, UK; 5 Centre for Biostatistics, School of Health Sciences, Faculty of Biology, Medicine and Health, University of Manchester, Manchester Academic Health Science Centre, Manchester, UK; 6 Faculty of Health, Social Care and Medicine, Edge Hill University Medical School, Ormskirk, UK

**Keywords:** Prognosis, Orthopaedic & trauma surgery, REGISTRIES

## Abstract

**Introduction:**

Understanding the prognostic factors associated with the failure of total elbow replacement (TER) is crucial for informing patients about risks and enabling shared decision-making regarding TER as a definitive management option. This protocol outlines the planned analysis of National Joint Registry (NJR) data to investigate prognostic factors for TER failure.

**Methods and analysis:**

The primary analysis will use the NJR elbow dataset, including all eligible patients who underwent TER surgery between April 2012 and December 2023. To incorporate ethnicity and comorbidities as potential prognostic factors, the NJR will be linked to the National Health Service (NHS) England Hospital Episode Statistics-Admitted Patient Care (HES-APC) data for a secondary analysis. The analysis will adhere to the REporting recommendations for tumour MARKer prognostic studies guidelines. The primary outcome under investigation is TER failure, defined as requiring revision surgery. Initially, the overall prognosis of TER will be examined using unadjusted net implant failure via the Kaplan-Meier method. The list of potential prognostic factors to be investigated in this study has been informed by a systematic review on this topic, input from patient and public involvement and engagement (PPIE) groups and a survey shared with healthcare professionals providing TER services. The relationship between each potential prognostic factor and failure will be assessed using univariable regression methods. Based on the findings from our systematic review, the univariable association will also be adjusted for age, sex and indication for TER surgery using multivariable regression methods. The extent of missing data will be reported, and the reasons for missing data will be explored. A very high degree of data completeness is expected, and a complete case analysis will be performed as the primary analysis. Multiple imputations will be considered as a sensitivity analysis.

**Ethics and dissemination:**

The NJR research committee approved this analysis, and the NHS Health Research Authority tool guidance dictates that the secondary use of such data for research does not require approval from a research ethics committee. The results from this analysis will be published in a peer-reviewed journal and presented at scientific conferences.

**Trial registration number:**

NCT06760585.

**STRENGTHS AND LIMITATIONS OF THIS STUDY**

- This is a protocol for the first analysis of prognostic factors associated with total elbow replacement failure using the National Joint Registry (NJR) elbow dataset, which is the largest elbow registry worldwide.
- This analysis will follow the methodological advances in prognostic factor research as proposed by the PROGnosis RESearch Strategy. It will be reported in accordance with the REporting recommendations for tumour MARKer prognostic studies guidelines.
- This analysis may be limited by missing prognostic factor data from the NJR and incomplete data linkage with the Hospital Episode Statistics-Admitted Patient Care dataset, as only NHS-funded procedures are included in this dataset.

## Introduction

 Total elbow replacement (TER) is a well-established treatment for painful arthritic elbows and is also used in cases of severe distal humerus fractures.^1^ TER, however, has been reported to have higher failure rates compared with other total joint replacements. It has been reported to have a 5-year failure rate of 6–10% and a 10-year failure rate of 15–19%, whereas total hip replacements have shown a 3% 5-year failure rate and a <5% 10-year failure rate.^1^,^8^ TER failure is commonly defined as the need for further elbow surgery involving the TER construct, which is widely known as revision surgery, where an implant is added, modified or removed. Revision surgery is linked to higher complication rates and poorer function than primary TER procedures.^9^

When examining factors associated with primary TER failure, a prognostic factor is defined as any variable that is associated with failure.^10 11^ Several existing studies have explored potential prognostic factors associated with the risk of failure in TER.^212^,^18^ However, a recent systematic review concluded that the current evidence on prognostic factors associated with the failure of TER is of low quality and is subject to significant risk of bias.^19^

Understanding the factors associated with TER failure could help to inform patients of the variable risks of failure and, therefore, aid decisions on whether to proceed with TER as a definitive management option.^20^ Improved understanding of prognostic factors may also reduce the need for TER revisions by enhancing treatment selection and identifying areas for innovative interventions.^10 21^ This information could also be used by government and health policymakers to identify patients at high risk, plan resource allocation and may help inform risk adjustment for monitoring hospital and surgeon performance.^10^ The James Lind Alliance, in their Priority Setting Partnership for elbow conditions, ranked research investigating the factors that affect the outcome and longevity of elbow replacements as the second most important research question in elbow conditions.^22^

Although investigating the prognostic factors associated with TER failure does not provide absolute risk prediction of failure, it could pave the way for future research to develop a prognostic model in TER that could be used for calculating the risk of TER failure in individual patients using a number of prognostic factors. That is beyond the scope of this research project.

This protocol describes a proposed analysis of the National Joint Registry (NJR) elbow dataset which aims to evaluate potential prognostic factors for TER failure. The NJR currently contains the largest elbow registry dataset in the world, which has been shown to have high completeness and accuracy.^23^ In order to incorporate additional potential prognostic factors not captured in the NJR, this study will combine the NJR elbow dataset with the NHS England Hospital Episode Statistics-Admitted Patient Care (HES-APC) data. Understanding prognostic factors is a crucial step for informing the development of strategies aimed at minimising the risk of failure and the necessity for additional surgery, which poses a burden on patients and society.^24^

## Methods and analysis

### Study design

This is a registry analysis using data from the NJR. The analysis is registered at clinicaltrials.gov (NCT06760585) and will adhere to the published guidance and recommendations on how to conduct prognostic factor research provided by the PROGnosis REsearch Strategy.^10^ This analysis will be reported following the REporting recommendations for tumour MARKer prognostic studies (REMARK) guidelines.^25^ While REMARK was developed as the reporting guideline for prognostic factor studies in oncology, it has now been widely adopted outside oncology. Experts in prognosis research have recommended their use.^10^

### Patients and public involvement

The analysis objective was developed in collaboration with the patient and public involvement and engagement (PPIE) group at Wrightington Hospital. All PPIE members emphasised the importance of investigating how long a TER is likely to last (overall prognosis) and identifying the factors that may affect the outcomes (prognostic factors). The group also contributed to a list of potential prognostic factors that are important to patients. In terms of the outcomes under investigation, PPIE members stressed the significance of TER failure, which necessitates revision surgery, as a critical outcome. The ongoing involvement of the PPIE team will help ensure that the results from this analysis are shared appropriately with patients and clinicians.

### Data sources

The analysis will use NJR data from April 2012 to December 2023. Furthermore, this dataset will be linked to the NHS England HES-APC data to supplement patient information that is not collected by the NJR, including patient comorbidities and ethnicity. The HES-APC data available for linkage only extends to 31 March 2022; therefore, the linked data will be used for a subgroup analysis. Mortality outcomes will be collected by linking the NJR data to the Civil Registrations of Death dataset. If death occurs before the revision outcome, it will be used to censor patients.

The NJR commenced the collection of data on elbow replacement procedures in April 2012. It is mandatory for all NHS and private hospitals in England, Wales, Northern Ireland, the Isle of Man and the States of Guernsey to submit data on elbow replacement surgeries, including both primary and revision procedures, to the NJR.^26^ The NJR, the British Orthopaedic Trainee Association, the British Orthopaedic Association, British Elbow and Shoulder Society (BESS) and the Royal College of Surgeons of England audited the NJR dataset in England. The TER dataset exhibited a completeness rate of 94% and an accuracy rate of over 97%.^23^ The data collected by the NJR have undergone various changes since the onset of elbow data collection, and these changes are detailed in online supplemental file 1.

The HES-APC data will be linked to the NJR data using an established process that is routinely used by NJR and detailed in a previous publication.^27^ For procedures to be linked, the start date of the HES-APC hospital episode must be the same as or before the NJR operation date, and the NJR operation date must be before or on the same day as the end date of the HES-APC episode. Access to the data and data linkage is facilitated under NJR permissions as explained in the Ethics and dissemination section.

### Participants and eligibility criteria

All patients who have given consent and have a primary TER on the NJR elbow dataset will be included in this analysis. Patients will be excluded if it is not possible to trace them after surgery, if their ID numbers are invalid or if the surgery was not performed in England.

Procedures will be excluded for the following inconsistencies in the data: (1) if a revision TER occurs before a primary TER on the same side; (2) if the procedure type reported to the NJR does not match the implant components submitted.

Eligible NJR procedures will be linked to the available HES-APC data, which include all patients’ hospital episodes from 23 October 1996 to 31 March 2022. Hospital episodes with invalid ID numbers, implausible dates or unknown discharge dates will be excluded, as will duplicate episodes.

### Outcome measure

The primary outcome in this analysis will be the failure of TER, as indicated by the need for revision surgery recorded on the NJR. The NJR’s definition of ‘revision surgery’ will be used, which encompasses any operation involving the addition, removal or modification of one or more components in a joint replacement.^28^ This includes debridement, antibiotics and implant retention with modular exchange procedures.

The NJR elbow dataset reliably collects data on revision outcomes. Patients with joint replacements listed in the NJR are identified using national tracing services. In England and Wales, this is carried out through the NHS England’s Data Access Tracing Service. A unique national identifier is generated to link all primary and revision procedures for the same joint in a single patient. The success rate of tracing patients with joint replacements in the NJR has consistently been above 94% since 2003.^29^ Moreover, a recent national audit demonstrated high data completeness (94%) for revision TER procedures.^23^

The NJR and HES-APC data are limited by the lack of patient-reported outcomes (PROMs) as well as clinical and functional outcomes. Furthermore, it is difficult to ascertain whether patients underwent additional non-revision surgeries on the elbow. This is, therefore, a limitation of this study. These shortcomings underscore the necessity for a structured approach to collecting elbow replacement PROMs, similar to the established practices for shoulder, hip and knee replacements.

### Prognostic factors to be investigated

The process of selecting which potential prognostic factors to be evaluated involved conducting a systematic review to appraise existing evidence on prognostic factors associated with TER failure,^19^ collaborating with the PPIE group at Wrightington Hospital, administering a survey to communities of healthcare professionals and using the clinical expertise of the authors. The data quality for each factor will be assessed before finalising the selection for analysis. We identified 27 factors from the NJR dataset that merit investigation as potential prognostic factors and 4 from the HES-APC dataset. This includes 14 factors previously studied and 17 unexplored factors (table 1).

**Table 1:** The list of potential prognostic factors that are available to be investigated on the NJR and HES-APC

Status	Type of factor	Potential prognostic factor	Data source	How are the data collected?	Data type
Factors previously investigated in the literature	Patient-related factors	Age	NJR	Indirectly derived from NJR data	Continuous
		Sex	NJR	Directly submitted to NJR	Categorical
		BMI	NJR	Directly submitted to NJR	Continuous
		Dominant arm	NJR	Indirectly derived from NJR data	Categorical
		Indication for surgery	NJR	Directly submitted to NJR	Categorical
		Ethnicity	HES-APC	Codes from medical records	Categorical
	Implant-related factors	Fixation type	NJR	Directly submitted to NJR	Categorical
		Implant linkage	NJR	Derived from the implant codes of submitted implants	Categorical
		Implant type	NJR	Derived from the implant codes of submitted implants	Categorical
		If RHR was used	NJR	Derived from the implant codes of submitted implants	Categorical
		Implant stem substrate	NJR	Derived from the implant codes of submitted implants	Categorical
		Implant stem finish	NJR	Derived from the implant codes of submitted implants	Categorical
	Surgical-related factors	Grade of primary surgeon	NJR	Directly submitted to NJR	Categorical
		Year of surgery	NJR	Directly submitted to NJR	Continuous
Factors not investigated in the literature	Patient-related factors	Weight	NJR	Directly submitted to NJR	Continuous
		Height	NJR	Directly submitted to NJR	Continuous
		ASA	NJR	Directly submitted to NJR	Categorical
		Socioeconomic status	NJR	Indirectly derived from NJR data	Categorical
		Comorbidities (osteoporosis)	HES-APC	ICD-10 codes on HES data	Categorical
		Comorbidities (diabetes)	HES-APC	ICD-10 codes on HES data	Categorical
		Comorbidities indices	HES-APC	ICD-10 codes on HES data	Continuous
	Implant-related factors	Cement type	NJR	Derived from the implant codes of submitted implants	Categorical
		Polyethylene type	NJR	Derived from the implant codes of submitted implants	Categorical
		Presence of anterior flange	NJR	Derived from the implant codes of submitted implants	Categorical
	Surgical-related factors	Funding type	NJR	Directly submitted to NJR	Categorical
		Hospital type	NJR	Indirectly derived from NJR data	Categorical
		Humeral bone grafting	NJR	Directly submitted to NJR	Categorical
		Ulnar bone grafting	NJR	Directly submitted to NJR	Categorical
		Intraoperative complications	NJR	Directly submitted to NJR	Categorical
		Surgical approach	NJR	Directly submitted to NJR	Categorical
		Region where TER was performed	NJR	Indirectly derived from NJR data	Categorical

ASA, American Society of Anaesthesiologists; BMI, Body Mass Index; HES-APC, Hospital Episode Statistics-Admitted Patient Care; ICD-10, International Classification of Diseases, 10th Revision; NJR, National Joint Registry; RHR, radial head replacement; TER, total elbow replacement.

It is important to highlight that both patients and healthcare professionals identified the volume of surgeries performed by specific surgeons and hospitals as an important potential prognostic factor that warrants further investigation. Although previous studies have explored the relationship between surgeon and hospital volume in relation to hip and shoulder replacements,^30 31^ the statistical modelling used in these analyses is complex and beyond the scope of this study. Therefore, a separate study should be conducted to examine this relationship in more detail.

The use of HES-APC data enables the investigation of comorbidities as potential prognostic factors for the first time. We will evaluate both the Charlson Comorbidity Index and the Elixhauser Comorbidity Index. The Elixhauser Comorbidity Index encompasses a wider range of comorbid conditions and is reported to more accurately predict inpatient mortality following orthopaedic surgery compared with the Charlson Comorbidity Index.^32^ However, both the NJR and HES-APC data lack information on specific conditions related to the elbow, such as stiffness, functional weakness and ulnar neuropathy. These conditions are considered important by healthcare professionals who participated in the survey and would require further investigation in a separate study using different datasets.

The collection of TER procedures on the NJR was performed using the NJR data collection tool known as the Minimal Data Set (MDS). Since the start of TER data collection, there have been 4 MDS versions, V.5–V.8. In this analysis, categorical variables will be classified based on the categories stated in the latest MDS form (MDS V.8). The measurement of each prognostic factor, including the categories to be used, is summarised in tables2^4^.

**Table 2:** How patient-related prognostic factors will be measured

Variable	Data used in primary analysis	Data used in planned sensitivity analysis
Age	Continuous: measured in years	Categorical: ≥60/<60
Sex	Categorical: male/female/indeterminate	No difference
BMI	Continuous	Categorical: underweight/normal/overweight/obese/morbidly obese^41^
Height	Continuous: measured in centimetres	No difference
Weight	Continuous: measured in kilograms	No difference
Dominant arm	Categorical: yes/noPatients with unknown category will be excluded	Categorical: yes/no
ASA	Categorical: ASA1–ASA5	No difference
Indication for surgery	Categorical: Trauma/elective. Acute trauma/inflammatory/trauma sequelae/osteoarthritis/other.	No difference
Socioeconomic status	Categorical: Index of multiple deprivation quintile (V.2015)	No difference
Ethnicity	Categorical: classification is based on the ONS group classification 6a^42^Asian (includes Asian British or Asian Welsh)/black (includes black British, black Welsh, Caribbean or African)/white/mixed or multiple ethnicity/other ethnic group	No difference
Comorbidities (diabetes)	Categorical: yes/no	No difference
Comorbidities (osteoporosis)	Categorical: yes/no	No difference
Comorbidities indices	Continuous: CCI with revised weightings developed by Dr Foster Intelligence and used by the Health and Social Care Information Centre as part of the SHMI. Elixhauser Comorbidity Index.	No difference

ASA, American Society of Anaesthesiologists; BMI, Body Mass Index; CCI, Charlson Comorbidity Index; ONS, Office for National Statistics; SHMI, Summary Hospital-level Mortality Indicator.

**Table 3:** How implant-related prognostic factors will be measured

Variable	Data used in primary analysis	Data used in planned sensitivity analysis
Fixation type	Categorical: cemented/uncemented/hybrid	No difference
Implant linkage	Categorical: linked/unlinked	No difference
Implant type	Categorical: Coonrad-Morrey/discovery/latitude legacy/latitude EV/latitude hybrid/GSB III/MUTARS/Nexel/IBP	No difference
If RHR was used	Categorical: yes/no	No difference
Implant stem substrate	Categorical: cobalt chrome/titanium/stainless steel	No difference
Anterior flange	Categorical: yes/no	No difference
Implant stem finish	Categorical: plasma-sprayed/PMMA-coated/beaded porous-coated	No difference
Polyethylene type	Categorical: UHMWPE/HXLPE/vitamin E-HXLPE	No difference
Cement type	Categorical: Low/medium/high viscosity Antibiotic-loaded/no antibiotics	No difference

EV, EVolution; GSB III, Gschwend-Scheier-Bähler III; HXLPE, highly cross-linked polyethylene; IBP, Instrumented Bone Preserving; MUTARS, Modular Universal Tumour and Revision System; PMMA, polymethylmethacrylate; RHR, radial head replacement; UHMWPE, ultra-high molecular weight polyethylene.

**Table 4:** How surgical related prognostic factors will be measured

Variable	Data used in primary analysis	Data used in planned sensitivity analysis
Funding	Categorical: NHS/independent sector	No difference
Hospital type	Categorical: NHS/independent sector	No difference
Grade of primary surgeon	Categorical: consultant/other	Categorical: consultant/SpR/senior fellow/F1-ST2/specialty doctor/other
Region	Categorical: East of England/London/Midlands/North East and Yorkshire/North West/South East/South West	No difference
Year of surgery	Continuous/discrete: the year of surgery	No difference
Humeral bone grafting	Categorical: yes/no	No difference
Ulnar bone grafting	Categorical: yes/no	No difference
Approach	Categorical: posterior/lateral	No difference
Intraoperative complications	Categorical: none/bone penetration/fractures/neurovascular-related complication/other	Categorical: None/humeral shaft penetration/ulna shaft penetration/humerus fracture/ulna fracture/nerve injury/vascular injury/other. None/fracture or bone penetration/nerve injury/vascular injury/other.

F1, Foundation Year 1; NHS, The National Health Service; SpR, Specialist Registrar.

Most of the information pertaining to potential prognostic factors is directly submitted using the MDS forms. Additionally, several potential prognostic factors are indirectly inferred from this data, which are detailed below along with the method used to derive the data:

- Age will be calculated using the date of birth and date of surgery.
- The dominant arm will be established using the dominant hand as submitted to NJR (left, right, ambidextrous or unknown) and which side has been reported to have undergone TER.
- Implant information will be derived from the implant components submitted to the NJR using the same methods used to classify TER implants in the NJR annual report.
- Socioeconomic status will be derived from postcode as an Index of Multiple Deprivation quintile (V.2015).^33^
- Comorbidities will be derived from the ICD-10 (International Classification of Diseases, 10th Revision) codes reported by HES-APC. The list of comorbidities for each patient will include all comorbidities available in the HES-APC hospital episodes up to and including the primary TER.
- Comorbidity indices will be calculated using comorbidities derived from the ICD-10 codes reported by HES-APC and using methods described by Penfold *et al*.^34^

### Statistical analysis

#### Descriptive analysis

Descriptive analyses of all the available variables will be undertaken initially. Frequencies and percentages will be used to describe categorical variables. The distribution of continuous variables will be assessed using histograms and summarised using the mean and SD or median and IQRs.

#### Overall prognosis

Unadjusted net implant failure will be estimated using the Kaplan-Meier method. Unrevised TERs are either censored on 31 December 2023 or the date of death, whichever is the earliest.

#### Statistical modelling methods

Initially, the relationship between each potential prognostic factor and the failure of TER will be examined using univariable Cox regression methods. After that, we will investigate this relationship while adjusting for other prominent variables that may affect this association by using multivariable Cox regression modelling methods. Based on the results of our systematic review,^35^ we will adjust for age, sex and the indication for TER surgery.

Hazard ratios from univariable (ie, unadjusted) and multivariable (ie, adjusted) regression methods will be estimated for each prognostic factor. Flexible parametric models will be considered if the Cox proportional hazard assumptions are violated.^36^ For continuous predictors, non-linear associations between the outcome and predictors will be explored using fractional polynomial methods.

#### Sample size

We plan to use the complete elbow dataset from the NJR for this analysis; therefore, no formal sample size calculation will be performed as the total sample size and the number of failures requiring revision have already been predetermined. We will refer to Schmoor’s sample size calculation guidance to determine the number of predictor parameters we can include in our regression modelling strategies while limiting the potential for overfitting.^37^ Based on our previous study using the NJR elbow dataset, which included all TERs between 1 April 2012 and 31 December 2022,^38^ we expect the dataset for the analysis to include more than 3891 TERs, with over 244 of them requiring revision.

#### Missing data

Most of the potential prognostic factors collected by the NJR are compulsory for hospitals to submit and so are unlikely to be missing. Body Mass Index (BMI) and hand dominance do not have to be reported to the NJR if the data are not available, and the collection of BMI for elbows only started with the use of MDS V.7. This is likely to lead to missing data for those two factors. The extent of missing data will be reported, and the likely reasons for missing data will be explored.

Due to the expected high degree of data completeness, it is intended that the primary analysis for each prognostic factor will be based on a complete case analysis. We will also explore the use of multiple imputations as sensitivity analyses, where appropriate. A multivariate multiple imputation model, which includes the TER failure outcome and other prognostic factors, will be implemented. This approach avoids excluding participants from the analysis and is an effective method of handling missing prognostic factor information.^36^

#### Sensitivity analysis

In the primary analysis, several potential prognostic factors—including age and BMI—will be analysed as continuous variables. In the sensitivity analysis, these factors will be treated as categorical variables. This approach is motivated by the use of categorical data in previous literature, allowing for comparison with past studies and the potential combination of results in meta-analyses.

In addition, other prognostic factors such as intraoperative complications, grade of primary surgeon, arm dominance and diabetes as comorbidity will be categorised differently in the sensitivity analysis compared with the primary analysis. The specific categories for the sensitivity analysis are listed in tables2^4^.

The HES-APC dataset includes only NHS-funded procedures. If a significant number of procedures are conducted in the independent sector and, therefore, cannot be linked with HES-APC, a sensitivity analysis will be performed to compare those included in HES-APC with those carried out in the independent sector.

## Ethics and dissemination

This analysis will use pseudonymised data that are anonymous to the researchers who use it. Patients consented to inclusion in the NJR according to standard practice, with permission under the Health Service (Control of Patient Information) Regulations, otherwise referred to as Section 251 support.^39^ The NJR Research Committee approved this analysis,^40^ and the NHS Health Research Authority tool guidance dictates that the secondary use of such data for research does not require approval from a research ethics committee.^40^ The results of this analysis will be published in a peer-reviewed journal and presented at scientific conferences.

## Supplementary material

10.1136/bmjopen-2025-098729 online supplemental file 1
