# Supplementary material for: Prognostic factors associated with failure of total elbow replacement: a protocol for analysis of National Joint Registry data in England
Source: BMJ Open. 2025 Jul 1;15(7):e098729. doi: 10.1136/bmjopen-2025-098729 (PMC12215097; doi:10.1136/bmjopen-2025-098729)
Supplement: online supplemental file 1 [file bmjopen-15-7-s001.docx]

**Supplementary File 1**

**The changes in NJR data collection between different MDS version**

Minimal Data Set (MDS) version 5 was used between April 2012 and November 2014, MDS Version 6 was used between November 2014 and June 2018, and MDS Version 7 was used between June 2018 and June 2023.

| **Variable** | **MDS** | **Changes in MDS** | **How will changes be addressed in this study** |
| --- | --- | --- | --- |
| Sex | 5-7 | None |  |
| BMI | 7 | Only started data collection in MDSv7 | 1. Use available BMI data 2. Calculate BMI from height and weight if available |
| Dominant hand | 5-7 | None |  |
| ASA | 5-7 | None |  |
| Funding | 5-7 | None |  |
| Grade of surgeon | 5-7 | None |  |
| Assistant | 5-7 | None |  |
| Side | 5-7 | None |  |
| Indication | 5-7 | MDSv5 had a failed humeral hemiarthroplasty as an indication for primary TER. This has changed to revision TER since MDSv6. | Primary TER performed humeral hemiarthroplasty will be excluded as this is a revision procedure |
| Surgical Approach | 5-7 | None |  |
| Anaesthetic type | 5-7 | MDSv5 and MDSv6 had four categories: General/ Regional – Nerve block / Regional Epidural / Regional – Spinal  MDSv7 has only two categories general/ regional nerve block | Not reported in this study |
| Minimally invasive | 5-7 | None |  |
| Computer guided surgery | 5-7 | None |  |
| Chemical VTE prophylaxis | 5-7 | Addition of Factor Xa Inhibitor (e.g. Rivaroxaban/Apixaban) to MDSv6 and MDSv7 | Not reported in this study |
| Mechanical VTE prophylaxis | 5-7 | None |  |
| Humeral bone grafting | 5-7 | Type of Bone graft was added in MDS 7 | Not reported in this study |
| Ulna bone grafting | 5-7 | Type of Bone graft was added in MDS 7 | Not reported in this study |
| Intra-operative complications | 5-7 | None |  |
| Fixation type | 5-7 | None |  |
| Approach | 5-7 | None |  |
