# Supplementary material for: Prognostic factors associated with failure of total elbow replacement: a protocol for analysis of National Joint Registry data in England
Source: BMJ Open. 2025 Jul 1;15(7):e098729. doi: 10.1136/bmjopen-2025-098729 (PMC12215097; doi:10.1136/bmjopen-2025-098729)
Supplement: Supplementary file 2 [file bmjopen-2025-098729.reviewer_comments.pdf]

PEER REVIEW HISTORY

BMJ Open publishes all reviews undertaken for accepted manuscripts. Reviewers are asked to complete a checklist review form and are provided with free text boxes to elaborate on their assessment. These free text comments are reproduced below.

ARTICLE DETAILS

Title (Provisional)

Prognostic factors associated with failure of total elbow replacement: a protocol for analysis of National Joint Registry data in England

Authors

Hamoodi, Zaid; Sayers, Adrian; Whitehouse, Michael R; Kearsley-Fleet, Lianne; Sergeant, Jamie C; Watts, Adam C

VERSION 1 - REVIEW

**Reviewer**	**1**
**Name**	**Gill, Stephen**
**Affiliation**	**Barwon Health and St John of God Hospital, Barwon Centre for Orthopaedic Research and Education (B-CORE)**
**Date**	**08-Apr-2025**
**COI**	**None**

This is a methodologically sound protocol written by a suitably expert team. The importance of the research is supported by James Lind Alliance. Considerable effort has been undertaken to establish potential prognostic factors associated with TER failure. I wish the authors well in conducting the study and look forward to the dissemination of results.

I have a few minor comments for consideration:

Tumour MARKer prognostic studies (REMARK) guidelines – consider providing rationale for using these guidelines.

Can you report the proportion NHS v private operations in the dataset, with consideration of the implications for data matching to HES-APC data?

Will need to fix a few typo's and drafting remnants throughout the document.

**Reviewer**	**2**
**Name**	**Lambert, Simon**

Affiliation **University College London Hospitals NHS Foundation Trust, Department of Trauma and Orthopaedic Surgery**

Date **04-May-2025**

COI **None**

This study has been designed to assess the relative importance of factors associated with the outcome of Total Elbow Replacement (TER) performed in England, using data available from the English NJR for TER between 2012 and 2023. The goal is to define those factors which appear to be prognostic of outcome, with the end-point defined as revision of the TER for reasons associated with the implants.

The authors have published useful background articles on the subject, which are referenced in the bibliography. While the subject matter of the preceding publications overlaps with the present protocol, the aims of each of the previous studies has been discrete and clearly defined, and thus the present study is not a replication of work but an extension of previous work utilising the NJR and HES data for TER.

There are four comments which I hope are helpful:

1. There are minor typos in the caption for the Supplementary File 1 which could be helpfully reviewed.

2. The use of the REMARK guidelines could be usefully explained. It is not immediately apparent (unless the original REMARK guidelines are accessed and comprehended - which this reviewer did) why this set of guidelines was utilised for the review of prognostic factors. A short comment as to why the REMARK guidelines, and how they were adapted, were chosen as the methodology for this study would be invaluable. The lists of prognostic factors and metadata appears to comply with this reporting standard.

3. The use of the Elixhauser Comorbidity Index, rather than the more commonly used Charlson Comorbidity Index, which is shorter and perhaps more readily completed, might be explained. Readers might not be familiar with the Elixhauser as opposed to the Charlson: a short explanatory note would again be invaluable.

4. The value of a TER is not only derived from the implantation of a secure, durable prosthesis. Many otherwise mechanically satisfactory implantations are associated with stiffness (loss of active range of functional motion); non-implant related pain; functional elbow weakness; and ulnar neuropathy causing sensorimotor disturbance of the hand. These outcomes are considered negatively by patients. The presence of any of these factors prior to the otherwise technically satisfactory implantation increases the likelihood of a poorer outcome after implantation, and the acquisition of any of these as a result of implantation is often permanent and (at least, partially) disabling. These factors are often related to the surgical approach used for the implantation. How will the authors determine and assess those prior factors that lead to a poor outcome for TER which are not recorded

on the NJR MDS versions 7/8 and which do not lead to implant revision (but may lead to further surgery)? While this is technically not 'missing data' (therefore fulfils the criteria of the REMARK guidelines) it is actually 'not recorded data' (although the HES resource may help here). This appears to be limitation of this study which has not been discussed, and it is pertinent to the purpose of the study. How will the authors deal with this aspect, please?

The manuscript is otherwise well-written, well-organised, and clear. It is timely, and will form a valuable resource for global comprehension of the benefits and potential pitfalls of TER.

VERSION 1 - AUTHOR RESPONSE

Comments from Reviewer 1 (Dr. Stephen Gill):

Dear Dr Gill,

Many thanks for your review and valuable feedback. We agreed with your suggestions and made changes to the manuscript as listed below.

Comment 1: Tumour MARKer prognostic studies (REMARK) guidelines – consider providing rationale for using these guidelines.

Response: We added the following text to the Study Design section to explain our rationale for using REMARK "While REMARK was developed as the reporting guidelines for prognostic factor studies in oncology, it has now been widely adopted outside oncology. Experts in prognosis research have recommended their use."

Comment 2: Can you report the proportion NHS v private operations in the dataset, with consideration of the implications for data matching to HES-APC data.

Response: We do not know the exact proportion of NHS vs Private operations in the dataset until the analysis has started. We have considered the implication for data matching to HES-APC and added the following statement to the sensitivity analysis section "The HES-APC dataset includes only NHS-funded procedures. If a significant number of procedures are conducted in the independent sector and therefore cannot be linked with HES-APC, a sensitivity analysis will be performed to compare those included in HES-APC with those carried out in the independent sector."

Comment 3: Will need to fix a few typos and drafting remnants throughout the document.

Response: Thank you for highlighting this. Several typos were noticed and corrected.

Comments from reviewer 2:

Dear Dr Lambert,

Many thanks for your review and valuable feedback. We agreed with your suggestions and made changes to the manuscript as listed below.

Comment 1: There are minor typos in the caption for the Supplementary File 1 which could be helpfully reviewed.

Response: thank you for highlighting this in Supplementary File 1. We have corrected them, and the current caption is "Minimal Data Set (MDS) version 5 was used between April 2012 and November 2014, MDS Version 6 was used between November 2014 and June 2018, and MDS Version 7 was used between June 2018 and June 2023."

Comment 2: The use of the REMARK guidelines could be usefully explained. It is not immediately apparent (unless the original REMARK guidelines are accessed and comprehended - which this reviewer did) why this set of guidelines was utilised for the review of prognostic factors. A short comment as to why the REMARK guidelines, and how they were adapted, were chosen as the methodology for this study would be invaluable. The lists of prognostic factors and metadata appear to comply with this reporting standard.

Response: We added the following text to the Study Design section to explain our rationale for using REMARK "While REMARK was developed as the reporting guidelines for prognostic factor studies in oncology, it has now been widely adopted outside oncology. Experts in prognosis research have recommended their use."

Comment 3: The use of the Elixhauser Comorbidity Index, rather than the more commonly used Charlson Comorbidity Index, which is shorter and perhaps more readily completed, might be explained. Readers might not be familiar with the Elixhauser as opposed to the Charlson: a short explanatory note would again be invaluable.

Response: statement added to the Prognostic factors to be investigated section "The Elixhauser Index encompasses a wider range of comorbid conditions and is reported to more accurately predict inpatient mortality following orthopaedic surgery compared to the Charlson Comorbidity Index."

Ref: Menendez ME, Neuhaus V, van Dijk CN, Ring D. The Elixhauser Comorbidity Method Outperforms the Charlson Index in Predicting Inpatient Death After Orthopaedic Surgery. Clin Orthop. 2014 Sep; 472 (9):2878–86. https://doi.org/10.1007/s11999-014-3686-7 PMID: 24867450

Comment 4: The value of a TER is not only derived from the implantation of a secure, durable prosthesis. Many otherwise mechanically satisfactory implantations are associated with stiffness (loss of active range of functional motion); non-implant related pain; functional elbow weakness; and ulnar neuropathy causing sensorimotor disturbance of the hand. These outcomes are considered negatively by patients. The presence of any of these factors prior to the otherwise technically satisfactory implantation increases the likelihood of a poorer outcome after implantation, and the acquisition of any of these as a result of implantation is often permanent and (at least, partially) disabling. These factors are often related to the surgical approach used for the implantation. How will the authors determine and assess those prior factors that lead to a poor outcome for TER which are not recorded on the NJR MDS versions 7/8 and which do not lead to implant revision (but may lead to further surgery)? While this is technically not 'missing data' (therefore fulfils the criteria of the REMARK guidelines) it is actually 'not recorded data' (although the HES resource may help here). This appears to be limitation of this study which has not been discussed, and it is pertinent to the purpose of the study. How will the authors deal with this aspect, please?

Response: We agree with your comment. As you pointed out, the NJR dataset does not contain this information. Additionally, the HES-APC dataset may contain some of this information but with uncertain reliability. Following statement added:

1) In the outcome measure section “The NJR and HES-APC data are limited by the lack of patient-reported outcomes (PROMs), as well as clinical and functional outcomes. Furthermore, it is difficult to ascertain whether patients underwent additional non-revision surgeries on the elbow. This is therefore a limitation of this study. These shortcomings underscore the necessity for a structured approach to collecting elbow replacement PROMs, similar to the established practices for shoulder, hip, and knee replacements.”

---

2) In the Prognostic factors to be investigated section “The use of HES-APC data enables the investigation of co-morbidities as potential prognostic factors for the first time. We will evaluate both the Charlson Comorbidity Index and the Elixhauser Comorbidity Index. The Elixhauser Index encompasses a wider range of comorbid conditions and is reported to more accurately predict inpatient mortality following orthopaedic surgery compared to the Charlson Index. However, both the NJR and HES-APC data lack information on specific conditions related to the elbow, such as stiffness, functional weakness, and ulnar

neuropathy. These conditions are considered important by healthcare professionals who participated in the survey and would require further investigation in a separate study using different datasets.”
